# Prisons at Home and Abroad

**Published:** 1892-05-28

**Authors:** 


### Prisons at Home and Abroad.

Although of late years much attention has been paid by all nations to the treatment of prisoners with a view to their ultimate reformation, the Howard Association, now twenty-five years old, must be regarded, like the great man whose name it bears, as a pioneer in the task. This gives a double weight to any of its official utterances, which is increased still further when an association which has always "leaned to mercy's side" criticises with marked severity the leniency shown to criminals by other nations. This is done not only in the annual report for 1891, but still more sharply in a pamphlet issued by the Association and entitled "The Collegiate and Hotel Prisons of the United States."

"Collegiate" and "hotel." These two words contain the accusation brought against the American prisons, and brought with great appearance of justice, though there are one or two points on which we think the condemnation is unduly severe. On the "hotel" question we quite agree with the writer of the pamphlet. Even in Elmira it seemed to us that the dietary was surprisingly luxurious; and Elmira is not a mere prison, but a reformatory for young men, who are there put through a course of education, mental and physical, implying a strain which must be met by a sufficiency of well-chosen food. We do not forget either that the people of the United States are accustomed to a more varied and luxurious dietary than is usual with the corresponding classes here; and that they naturally incline to feed even their prisoners better than the tenants of Woking and Pentonville. But in no country in the world that we know of can "honest poverty" get a daily meat diet, with a sufficiency of vegetables and coffee, without working for it. To manage this one must be an occupant of the California State Prison of Folsom. This is the fare of the lowest grade prisoners, who lounge about in full companionship with each other, and fulfil the old charwoman's aspiration of "doing nothing for ever and ever." If they work at the slate quarries they get in addition soup, tea and cake, and meat for supper. A yet higher grade get in addition chops or steaks for breakfast, fruit, and a dinner "worthy of a good hotel." In short, a well-behaved prisoner in Folsom Gaol lives more luxuriantly than the average middle-class citizen; and this without any thought of moral improvement resulting from physical indulgence. We can but echo the words of the pamphlet, that "this is not only outrageously unfair to the taxpayers, but offers strong temptation to the honest poor by inducing them to qualify for a convict life."

When, however, the condemnation is extended to the "collegiate" system, by which education is made an important part of the treatment of prisoners, we find ourselves completely at issue with the writer. It is not easy for an uneducated man to earn his living in these days, a man who has no trade, no special capacity. Oakum-picking does not command a high price in the labour market; stone breaking is not to be had in every place, and the treadmill, being of no economic value, has found no place outside the prison walls. Therefore, if you set free a thief with only these accomplishments in addition to his old trade, it is not difficult to predict which he will follow. Rouse his intellect, and you have a better chance of touching his conscience. Teach him a trade, and there is a possibility of his developing into an honest citizen. But it is objected, "considering the severe competition of honest industry, it is most unfair to the ordinary American workman that these criminals at Elmira should be taught artistic and fancy trades, such as modelling and designing from nature, embossing in brass, executing portraits in copper, telegraphy, and so forth." You cannot teach a man what it is not in him to learn; doubtless there are prisoners at Elmira who cannot be made into artists and telegraph operators by any means under the sun. But if they can, why should anyone grudge them the opportunity of learning? Are we to argue thus: "This man, with proper training, could earn a good living at a certain business. So could others, morally worthier than he; but I do not know them, and so cannot help them. But for their sakes I will withhold from this man, whom I could help, the instruction that he needs." In the name of Him who came to save sinners, we ask if this is to be the principle of our dealing with our erring brothers? Let us give every advantage of technical education to the honest and industrious that we can; but to refuse help to one because we cannot help all is suspiciously like burying a talent in a napkin.

True, the method is expensive, but human souls have their value. If the results are good we dare not grudge the cost. And that the results are good is not denied. Again we quote from the pamphlet which would condemn the Elmira system: "In many instances the industrial discipline at Elmira has tended to reform individuals. But in this connection it is specially to be observed that every man liberated from Elmira before the maximum term, *has a situation found for him* to enter upon on discharge. This is a great help to reformation, but it is quite distinct from the operation of discipline *inside* Elmira." We give the writer the full benefit of his italics, but we ask him two questions: (1) Would it be possible to find a situation for each prisoner if he did not know some trade? (2) Does not the fact that situations can be found imply that the Elmira men are reliable and satisfactory, or employers of labour would not be willing to take them? One can hardly condemn a system which takes 75 per cent. of those subjected to it out of the criminal class.

We might point out various inconsistencies in the pamphlet itself, though it consists of only four pages, and also between it and the report of the association which publishes it. "Imprisonment is at best a necessary evil. As far as possible it should be short and sharp." Yet shortly before it mentions as telling against Elmira, that a group of twenty-four prisoners "had only undergone an average detention of one year, six months, and one day." Moreover, the report declares that cellular imprisonment cannot be judiciously maintained for more than two years. After this, "it becomes absolutely necessary, for the preservation of both bodily and mental health, that a comparatively liberal diet, together with relaxed conditions of companionship, shall be permitted." Thus at the end of two years something approaching to the American system must, in the opinion of those who condemn it, be resorted to. The association argue from these premises that imprisonments should be "short and rigorous," but in view of the fact that every daily paper chronicles the reappearance of prisoners for the thirtieth, fiftieth, or hundredth time before our courts, we are compelled to think that these short imprisonments do little to prevent crime. That serious faults do exist in American prisons we admit; that too great laxity is no benefit to the prisoner and a distinct wrong to the community we emphatically affirm; but that mere penal measures are really deterrent we are equally ready to deny. Every influence, religious, industrial, and social, that can arouse the prisoner to, first, repentance, and then self-respect ought to be applied; always remembering, however, that in a population composed entirely of criminals we have no right to expect better results from these influences than are shown by society at large.

